# Temporal dynamics of trauma memory persistence

**DOI:** 10.1098/rsif.2023.0108

**Published:** 2023-06-07

**Authors:** Michael B. Bonsall, Emily A. Holmes

**Affiliations:** ^1^ Mathematical Ecology Research Group, Department of Biology, University of Oxford, Oxford OX1 3RB, UK; ^2^ St Peter's College, Oxford OX1 2DL, UK; ^3^ Department of Psychology, Uppsala University, Uppsala 751 42, Sweden

**Keywords:** intrusive memories, Markov chains, probability, reconsolidation, stochastic dynamics

## Abstract

Traumatic events lead to distressing memories, but such memories are made all the worse when they intrude to mind unbidden and recurrently. Intrusive memories and flashbacks after trauma are prominent in several mental disorders, including post-traumatic stress disorder and can persist for years. Critically, the reduction of intrusive memories provides a treatment target. While cognitive and descriptive models for psychological trauma exist, these lack formal quantitative structure and robust empirical validation. Here, using techniques from stochastic process theory, we develop a mechanistically driven, quantitative framework to extend understanding of the temporal dynamic processes of trauma memory. Our approach is to develop a probabilistic description of memory mechanisms to link to the broader goals of trauma treatment. We show how the marginal gains of treatments for intrusive memories can be enhanced as key properties (intervention strength and reminder strength) of the intervention and memory consolidation (probability memories are labile) vary. Parametrizing the framework with empirical data highlights that while emerging interventions to reduce occurrence of intrusive memories can be effective, counterintuitively, *weakening* multiple reactivation cues may help reduce intrusive memories more than would stronger cues. More broadly, the approach provides a quantitative framework for associating neural mechanisms of memory with broader cognitive processes.

## 1. Introduction

Traumatic events (such as physical or sexual assaults, disasters and war experiences) are widespread [1], causing significant distress and morbidity, and a range of mental disorders. Post-traumatic stress disorder (PTSD) is characterized by ‘recurrent, involuntary and intrusive distressing memories of the traumatic event(s)’ [2]. What is special about this form of memory is that it is not only highly emotional [3], but it is thrust into mind unexpectedly against one's will [4] and can persist for years: henceforth we referred to these as *intrusive memories*. For trauma survivors, forgetting trauma might be a long-term goal, but counterintuitively the deliberate recall of trauma memories is key in evidence-based psychological therapies [5]. One hypothesis is that under some circumstances recalling memories can temporarily return them to a malleable, labile state [6,7]. This can be achieved via a so-called ‘reminder cue’ where a simple stimulus (such as a word, a smell or a visualization) acts to reactivate memory into a labile form. Critically, during this labile period, memories may be altered/disrupted (or left uninterrupted), before reconsolidating back into long-term memory [8]. The fundamental idea that consolidated memory is not permanent [9] but could again become available to alteration over a finite time window following a reminder (inferred to initiate memory reactivation) is termed ‘memory reconsolidation’ [10–12]. Memory alteration following retrieval plus various pharmacological or behavioural interventions has been achieved [13–16], though not without controversies and challenges [17]. This process suggests potential for trauma treatment innovation with procedures designed to interfere with memory reconsolidation [18,19] and critically here to make these intrusive trauma memories become non-intrusive.

While psychological models for the implications of psychological trauma are reasonably well developed [20–23], these approaches often lack quantitative predictions. Conceptual models are underpinned by the idea that a key psychopathological form of trauma recall is characterized by intrusive memories, and advances in these conceptual models have focused on developing neural bases for the combination of inflexible involuntary memories with voluntary, flexible memory [24,25]. Relatedly, elsewhere, we have argued for a hierarchical mechacognitive framework in which neural mechanisms are embedded in cognitive processes for focal mental health symptoms [26,27].

**Figure 1.**
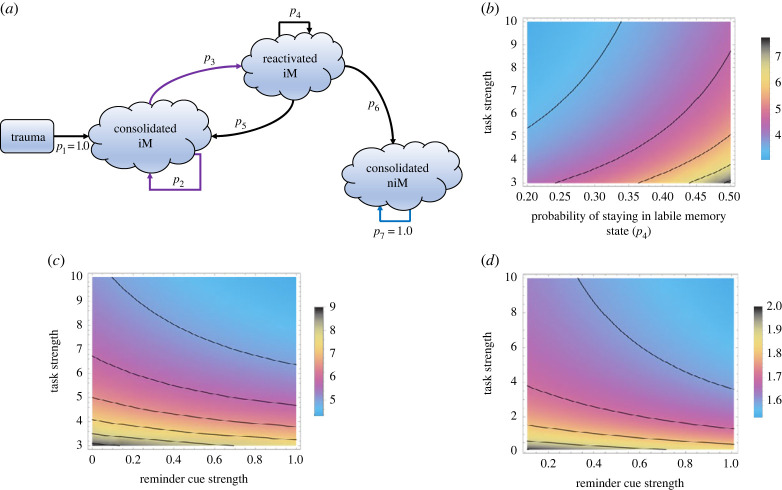
Persistence time of intrusive memories. (*a*) Schematic of the trauma model for different intrusive memory (iM) and non-intrusive memory (niM) states. Transitions are represented by different probabilities (*p*_1_–*p*_7_). Coloured arrows represent different rows in the transition matrix. (*b*) The effects of task strength and probability of memories staying reactivated (*p*_4_) maintaining intrusive memories in a reactivated state on persistence of intrusive memories. Expected time in the iM state increases as task strength weakens and/or probability of staying in the reactivated state increases. Beyond certain task strength, little further reduction of time in the iM state is achieved. (*c*) The effects of task strength and reminder cue strength on persistence of intrusive memories such that different combinations of task strength and reminder cue strengths minimize time in the iM state. (*d*) The effects of task strength and reminder cue strength on mixing times before memories absorb in the niM state such that different combinations of task strength and reminder cue strengths minimize time for memory to consolidate into the non-intrusive state. (Colours represent time in intrusive memory state).

To this end, here, together with empirical parametrization, we use a novel quantitative approach for investigating the temporal dynamics and persistence of intrusive memories after trauma within a memory reconsolidation framework. This framework uses probabilistic descriptors of transitions from one memory state to another. Here the processes of memory updating are described as a series of stochastic events culminating in the reconsolidation of a memory into a non-intrusive state. Our aim is to use this framework to describe how the intended reactivation of an intrusive memory (iM) via a reminder cue, followed by a behavioural task intervention can affect the probabilities of memories existing in different states. For modelling intrusive memories, our stochastic model is divided up into four distinct states (figure 1*a*): (i) initial trauma; (ii) consolidated iM; (iii) reactivated iM and (iv) non-intrusive form of memory (niM)—whereby a memory is rendered non-intrusive by the intervention.

Importantly, we define a set of probability transitions. These are the probability that after a traumatic event a given intrusive memory consolidates; here we assume that this always occurs (so *p*_1_ = 1.0) (but this need not be the case, see [26]), the probability that an intrusive memory, when spontaneously experienced, reconsolidates unaltered (*p*_2_), the probability that the intrusive memory is reactivated by a reminder cue (*p*_3_), the probability that memory stays in a reactivated state (allowing a time window for alteration) (*p*_4_), the probability that a reactivated memory reconsolidates as an intrusive memory and remains unaltered or is even strengthened (*p*_5_), the probability that the reactivated memory reconsolidates as a non-intrusive form of memory which is altered and weakened by the treatment intervention (*p*_6_) and the probability that the non-intrusive form of memory remains consolidated (so *p*_7_ = 1.0).

Critical to understanding how a trauma memory can be rendered non-intrusive is (i) that the intrusive memory can be reactivated with a reminder cue (*p*_3_) and (ii) that a task intervention can determine whether an intrusive memory reconsolidates in an altered form or not (*p*_5_). With this framework, it is then feasible to determine measures such as the expected time to absorption into the niM state, the expected intensity and the number of visits to the reactivated iM state before absorption into the niM state—all as a function of the task intervention, and/or the reminder cue.

## 2. Methods

### 2.1. Quantitative framework

To model intrusive memory temporal dynamics, we use a Markov chain approach. This aim of this framework is to capture the effects of an intervention (in our case a behavioural intervention; but the framework is equally applicable to pharmacological interventions) on intrusive memory (re)occurrence. Using this probabilistic model, memory states can be described as sequence of events in which the probability of transiting between states only depends on the state of the system at the previous event point. For modelling intrusive memories, we divide the Markov chain into four states: (i) prior trauma, no intrusive memory, (ii) a consolidated intrusive memory state, (iii) a reactivated intrusive memory and (iv) a non-intrusive memory state (see figure 1*a*). In matrix form this is represented by

2.1 $$P=\left(\begin{array}{cccc} 0 & \;p_{1}=1 & 0 & 0\\ 0 & \;p_{2} & \;p_{3} & 0\\ 0 & \;p_{5} & \;p_{4} & \;p_{6}\\ 0 & 0 & 0 & \;p_{7}=1 \end{array}\right),$$

where *p_i_* is the transition probability for the *i*th event. *p*_1_ is the probability that an intrusive memory consolidates and is laid down as a memory. For this version of our model, we assume that this always occurs. *p*_2_ is the probability that an intrusive memory reconsolidates and *p*_3_ is the probability that the intrusive memory is reactivated. *p*_4_ is the probability that reactivated memory stays reactivated, *p*_5_ is the probability that a reactivated memory reconsolidates as an intrusive memory and *p*_6_ is the probability that the reactivated memory reconsolidates as a niM. *p*_7_ is the probability that a niM remains in this state (here, we assume this in absorbing state so *p*_7_ = 1.0). Probabilities in each of the rows of the Markov chain sum to 1.

### 2.2. Model functions

Our aim is to understand how a behavioural task intervention and/or a reminder cue affect the probability of intrusive memories reconsolidating after reactivation into a non-intrusive state. This task intervention is described by its effects on the probability of a reactivated iM reconsolidating back into the iM state. In the matrix (equation (2.1)), this is probability transition *p*_5_ and, in a general form, we model this sort of task intervention as

2.2 $$p_{5}=\frac{1}{1+T}\;,$$

where *T* is the strength of the task intervention. As *T* increases the task intervention is more effective, *p*_5_ monotonically decreases and *p*_6_ increases (*p*_6_ = 1 – *p*_4_ – *p*_5_). We consider the role this form of task intervention has on influencing probabilistic outcomes of memory reconsolidation.

To describe the probability of reactivation (*p*_3_) following a reminder cue, we assume that this can be derived from a binary process where reactivation either does or does not occur. Probabilistically, this can be represented, in general form, as a logistic function,

2.3 $$p_{3}=\frac{1}{1+\exp(-\alpha )}\;,$$

where *α* is the strength of the reminder cue.

### 2.3. Analysis

Using this stochastic approach to model the (re)consolidation of intrusive memories, it is feasible to determine measures such as (i) the expected time to absorption into the niM state, (ii) number of visits to the reactivated iM state before absorption into the niM state and (iii) how long memories stay ‘mixed’ in different states—all as a function of task and/or reminder cue strength. This is achieved through analysis of the Markov chain (details in the electronic supplementary material) as the characteristic polynomial of a Markov chain (from Det(***P***−*λ***I**)) allows eigenvalues and (right) eigenvectors (**V**) to be determined. Using spectral decomposition yields an expression for the long-term probabilities of memory states: **P**^n^ = **V D**^n^**V**^−1^ where **D** is a diagonal matrix of eigenvalues. We develop this approach to analyse the temporal dynamics of intrusive memory reconsolidation, and further details on the analysis are given in the electronic supplementary material.

### 2.4. Numerics

For the numerical analysis and to investigate model predictions, we use the following formulations of the Markov chains and a canonical set of parameters.

To investigate persistence times (figure 1*b–d*), we use the following set of transition probabilities and parameter values: strength of reminder cue *α* = 1.0 and probability of reactivated memory staying reactivated *p*_4_ = 0.5.

To investigate the sensitivity of mixing times for memories, we use Latin hypercube sampling. Latin hypercube sampling is used to create random parameter sets with defined ranges for the probability memories remaining labile (*p*_4_: 0–1), task strength (*T*: 0–20) and reminder cue strength (*α*: 0–1). These parameter set combinations are used to re-evaluate memory mixing times (see electronic supplementary material, equations A17 and A18) and highlight how combination of parameters affects mixing times outcomes. Parameters that exhibit strong positive trends (in a scatter plot of mixing times against the parameter) suggest strong influence of high parameter values on memory mixing and persistence. Parameters with strong negative trends suggest strong influence of low parameter values on memory mixing and persistence. Whereas, a more random distribution would indicate a parameter value that has limited impact on memory mixing times. We use standard product-moment correlation coefficients to evaluate the influence of the parameter on mixing times.

**Figure 2.**
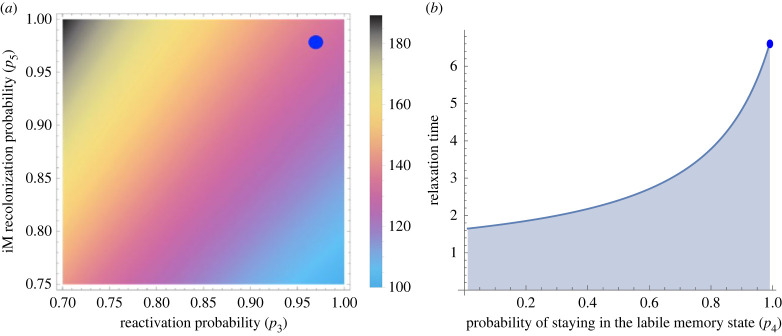
Stochastic trauma model predictions. Using the experimental data [28], analysis shows expected time in the intrusive memory (iM) state increases as reconsolidation probability (*p*_5_) increases and reactivation probability (*p*_3_) decreases. From the empirical parametrization of the unknown transition probabilities (*p*_2_ through to *p*_6_), the stochastic trauma model predicts (*a*) high reactivation and recolonization probabilities (blue dot) leading to intrusive memories that have low to intermediate persistence times in the consolidated iM state. (*b*) Time for memories to transit (so-called 'relaxation time') into the non-intrusive form of memory (niM) state have a limit (solid line) and for the experiments this time is expected to be low (blue dot).

To investigate persistence times (figure 2), we use the following set of transition matrices:

2.4 $$P=\left(\begin{array}{cccc} 0 & 1 & 0 & 0\\ 0 & 1-\frac{1}{1+e^{-\alpha }} & \frac{1}{1+e^{-\alpha }} & 0\\ 0 & \frac{1}{\left(1+T)(1+(1/(1+T))\right)} & \frac{\;p_{4}}{1+(1/(1+T))} & \frac{1-p_{4}}{1+(1/(1+T))}\\ 0 & 0 & 0 & 1 \end{array}\right),$$

where strength of reminder cue *α* = 0.5 and probability of memory staying reactivated *p*_4_ = 0.5, and

2.5 $$Q=\left(\begin{array}{cccc} 0 & 1 & 0 & 0\\ 0 & 0.5 & 0.5 & 0\\ 0 & 0.33 & 0.34 & 0.33\\ 0 & 0 & 0 & 1 \end{array}\right).$$

The initial memory state vector was *π* = [0,0.5,0.5,0]^T^. For the two tasks (*T*_1_, *T*_2_) acting multiplicatively, we use

2.6 $$P=\left(\begin{array}{cccc} 0 & 1 & 0 & 0\\ 0 & 1-\frac{1}{1+e^{-\alpha }} & \frac{1}{1+e^{-\alpha }} & 0\\ 0 & \frac{1}{\left(1+T_{1})(1+T_{2})(1+(1/(1+T_{1})(1+T_{2}))\right)} & \frac{\;p_{4}}{1+1/((1+T_{1})(1+T_{2}))} & \frac{1-p_{4}}{1+1/((1+T_{1})(1+T_{2}))}\\ 0 & 0 & 0 & 1 \end{array}\right),$$

with strength of reminder cue *α* = 0.5 and probability of reactivated memory staying reactivated *p*_4_ = 0.5. Initial memory states were *π* = [0,0.5,0.5,0]^T^.

**Figure 3.**
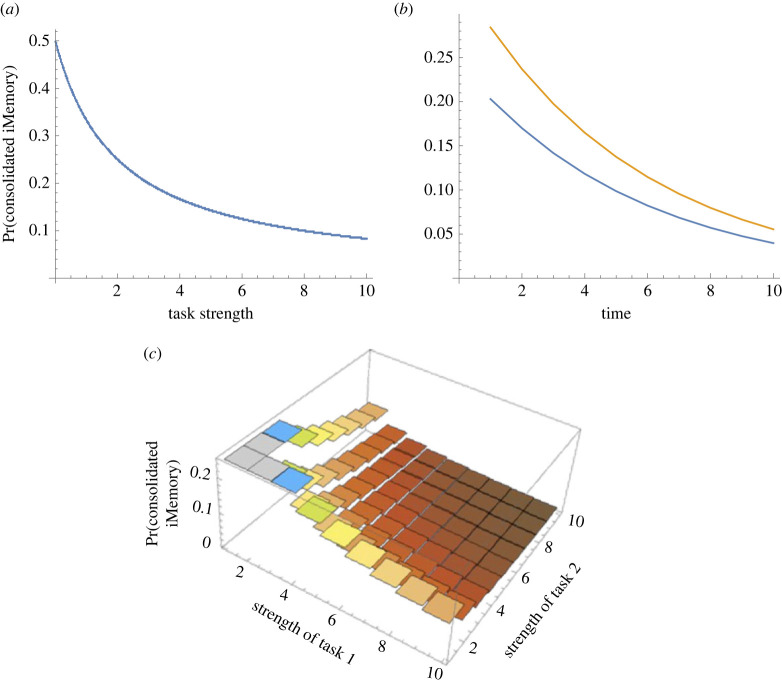
Simulation outcomes of trauma memory model for the effects of task strength on the probability of intrusive memories (iM). (*a*) Hypothesis: how does task strength affect the probability of reconsolidated intrusive memory by delivering one dose of a task (in the first time period)? Simulations reveal that the probability of intrusive memories declines for increasing task strengths. (*b*) Hypothesis: what is the role of task on the probability of intrusive memory reconsolidation over time by delivering one dose of task (in the first time period)? Simulations show that a task that interferes with the intrusive memory (blue line) is more likely to reduce the probability that intrusive memories reconsolidate compared with no task (orange line). (*c*) Hypothesis: what is the effect of multiple tasks on the probability of intrusive memory reconsolidation? Simulations reveal that combining more than one task (in the first time point), with certain task strengths, leads to stronger reduction in intrusive memories reconsolidating.

**Figure 4.**
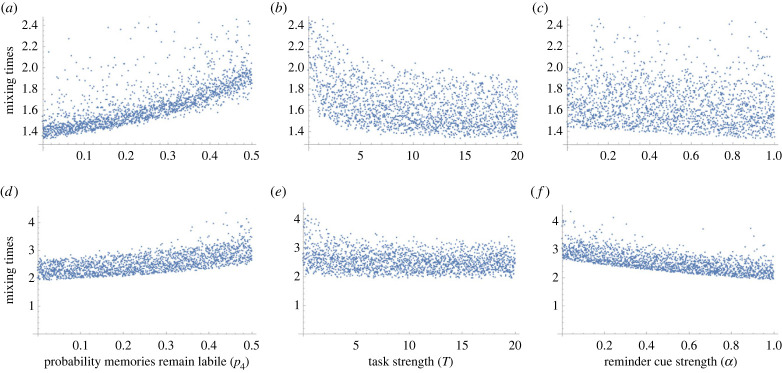
Mixing time (see electronic supplementary material, equations A17 and A18) responses for the Markov chain model (from Latin hypercube sampling) for (*a,d*) probability memories are maintained in a labile state (*p*_4_), (*b,e*) strength of the intervention task (*T*) and (*c,f*) strength of reminder rate (*α*). Rows represent different rates of baseline intrusive reactivation: (*a–c*) *p_3_* = 0.5; (*d–f*) *p_3_* = 0.05. With high levels of intrusive memory reactivation (*a–c*): (*p*_3_ = 0.5), there is (i) strong positive correlation of probability of maintaining memories in a labile state and maintaining mixed memory states (*ρ* = 0.605); (ii) strong negative correlation between task strength and maintaining mixed memory states (*ρ* = −0.428) and (iii) weak correlation with the strength of reactivation and mixing times (*ρ* = −0.143). By contrast, under weak levels of intrusive memory reactivation (*d–f*): (*p*_3_ = 0.05), while there is still strong positive correlation between memories being maintained in a labile state and mixing times (*ρ* = 0.636), the negative correlation between task strength and mixing times weakens (*ρ* = −0.186) and the negative correlation between reminder cue strength and mixing times strengthens (*ρ* = −0.692).

To investigate the effects of the multiplicative effects of the reminder cue (figure 3), we consider different scenarios with the transition matrices as for figure 4 (given above). For reminder cues which act independently, *p*_3_ = (1/(1 + exp(-*α*)))^*n*^, where *n* is the number of independent reminder cues (where *n* = 5, *α* = 0.5 and *p*_4_ = 0.5). For reminder cues that taper in magnitude in a conditional-dependent manner, we use a nested approach where *p*_3_ = 1/(1 + exp(−*p*_3_″)) with *p*_3_″ = 1/(1 + exp(−*p*_3_′)) and p_3_′ = 1/(1 + exp(−*α*)) (with *α* = 0.5 and *p*_4_ = 0.5). Initial memory states were *π* = [0,0.5,0.5,0]^T^.

Table 1 gives definitions of key terms and mathematical notation. All analyses were completed in Mathematica and the scripts are available at the Open Science Framework: https://osf.io/v4ynf/.

## 3. Results

### 3.1. Modelling intrusive memory dynamics

Analysis reveals that the expected time that memories remain in an intrusive state is dependent on the probability of maintaining a memory in the reactivated state (*p*_4_), and parameters associated with task strength and reminder cue strength (figure 1*b,c*). Expected time in the intrusive memory state increases as task strength weakens and/or the probability of memories being in a reactivated state increase. Of key importance, is that beyond a critical level of task strength, little further reduction of time in the intrusive memory state is achievable (figure 1*b*). Combinations of multiple task strengths can also minimize the time memories stay in the intrusive memory state (figure 1*c*).

Mixing time analysis determines how long it takes for memories to absorb into the non-intrusive memory state. As task strength and reminder cue strengths increase, mixing times are minimized before memories enter a non-intrusive form (figure 1*d*). Again, beyond critical combinations of task strength and reminder cue strength, little further minimization of mixing times is achievable. An upper bound on how quickly memories reach the non-intrusive state can be derived from an inequality analysis (details in the electronic supplementary material). This shows that the upper bound is critically determined by the probability of memories being held in the reactivated state (*p*_4_). High probability of memories remaining in the reactivated state can lead to long times before memories reach the absorbing state (non-intrusive memory state). The shape of this relationship suggests that there are limits beyond which any further balancing of memories being in the reactivated state leads to no further gains in how quickly memories move into the niM state.

### 3.2. Intrusive memory mixing times

Sensitivity analyses (using Latin hypercube sampling) reveal that mixing time (the time intrusive memories remain in a non-consolidated state—see electronic supplementary material, equations A17 and A18) is influenced by the probability that these memories remain in a labile state (*p*_4_), the strength of the intervention task (*T*) and the strength of the reminder cue (*α*) (figure 4). The importance of these different memory- and intervention-related processes depends on the rate at which memories are reactivated (*p*_3_). With high levels of intrusive memory reactivation (figure 4*a–c*), these intrusive memories will remain in a mixed state (i) by increasing the probability (*p_4_*) that the memories are in a labile state (correlation coefficient: *ρ* = 0.605) and (ii) as task strength (*T*) decreases (correlation coefficient: *ρ* = −0.428). Reminder cue strength (*α*) has a limited effect when background memory reactivation probability is high. By contrast, under weak levels of intrusive memory reactivation (figure 4*d–f*), while there are still strong positive effects of maintaining memories in a labile state on mixing times (correlation coefficient: *ρ* = 0.636), the effects of increasing task strength weakens (correlation coefficient: *ρ* = −0.186) and strengthening reminder cues decrease mixing times (correlation coefficient: *ρ* = −0.692).

### 3.3. Empirical parametrization

Critical to these Markov models are values for the transition probabilities. Once these are defined, we can use the stochastic model to evaluate the probability distributions of memories in different states over time, and assess the impact of various treatment interventions (see next section). In what follows in this section, we use empirical data to estimate canonical values for the parameters that underpin the transitions. In particular, we can parametrize the Markov model with experimental and/or clinical data in which intrusive memories have been manipulated [29]. To illustrate this approach, we use a dataset from an existing memory reactivation–reconsolidation study [28]. In this study to examine memory updating mechanisms to reduce the persistence of intrusive memories, participants viewed a film with traumatic content and recorded their intrusive memories to the film for 24 h (allowing the initial memory consolidation to occur). A day later, participants were randomized to one of four groups: no task control, memory reminder cue with task, task only or memory reminder cue only. The memory reminder cue was briefly viewing (2 s) film stills associated with specific intrusive memories. The task intervention involved playing the computer game Tetris® using mental rotation to optimize the placement of coloured blocks. Participants kept diaries of the number of intrusive memories over the subsequent week. From these diaries, estimates for the unknown probabilities (*p*_2_ to *p*_6_) in the transition matrix can be determined directly from empirical parametrization, assumptions about the distribution of intrusive memories and/or regression-based approaches (see electronic supplementary material).

Using assumptions that intrusive memories follow a discrete-valued Poisson distribution (see electronic supplementary material), the expected probabilities can be estimated using mean number of intrusive memories. As predicted, prior to intervention there is no significant difference in intrusive memories between participant groups in the 24 h following initial exposure to trauma stimuli (generalized linear model (GLM): *χ*^2^ = 0.834, d.f. = 3, *p* = 0.841), the mean number of intrusive memories is 3.334 (±0.268). For the iM state (where *p*_2_ + *p*_3_ = 1; see electronic supplementary material), the probability that intrusive memories reconsolidate unaltered (*p*_2_) is 1 − exp(−3.33) = 0.964 and hence the probability of reactivation (*p*_3_) is exp(−3.33) = 0.036.

From the diaries, the mean number of intrusive memories over the whole week is 5.111 (±0.996) for the no task control group, 1.889 (±0.411) for the memory reminder cue + task group, 3.83 (±0.682) for the task only group and 4.889 (±0.828) for the memory reminder cue only group. Again, using the discrete-valued Poisson distribution approach, the memory reminder cue group allows the probability that reactivated memory remains reactivated (*p*_4_) to be estimated, as this was the group to receive only the memory reminder cue. So, from this group the number of intrusive memories reflects memories in the reactivated state and the probability of no intrusive memories is 1 − exp(−4.889) = 0.9924. However, this probability combines *p*_3_ and *p*_4_ (as this group only had the reminder cue then recorded intrusive memories), so the marginal probability for *p*_4_ is the product of this joint probability and the probability of memory reactivation (*p*_4_ = *p*_3_ (*p*_4_ ∩ *p*_3_) = 0.035).

Similarly, from the memory reminder cue + task group, the probability that memories successfully reconsolidate into the non-intrusive memory state (*p*_6_) via the treatment intervention can be determined. From this group, the probability of no intrusive memories is exp(−1.889) = 0.151. Again, this is a combined probability of a reminder cue and a reconsolidation process (*p*_3_ and *p*_6_) so the marginal probability for *p*_6_ is the product of this joint probability and the probability of memory reactivation (*p*_6_ = *p*_3_ (*p*_6_ ∩ *p*_3_) = 0.005). Using information from the reactivated memory state that *p*_4_ + *p*_5_ + *p*_6_ = 1 (see electronic supplementary material), the probability that a reactivated memory reconsolidates as an intrusive memory (*p*_5_) is simply determined from 1− *p*_4_− *p*_6_ = 0.959.

With these transition probabilities, the stochastic model predicts low/intermediate persistence of iMs; this is principally driven by a combination of a high probability of intrusive memory reactivation with a high probability of intrusive memories reconsolidating in an unaltered way (figure 2*a*). Critically, this analysis suggests that while the task is effective, maintaining intrusive memories in a reactivated state (*p*_4_) is essential to allowing non-intrusive forms of the memory to be reconsolidated (see electronic supplementary material). The predicted long time to reach the non-intrusive memory state is constrained by a limit (figure 2*b*) preventing opportunities for further effective task interventions.

### 3.4. Simulating different treatment interventions

By definition, intrusive memories are those which come to mind involuntarily. The number of times a memory intrudes can be counted and recorded (say, in a diary). A reduction in the probability of the number of intrusions over a given time period is a primary outcome measure for recent intervention development [30–32]. Our stochastic framework can be used to simulate different treatment interventions. The expectation is that task memories (memories that are encoded during an intervention) interact and interfere with intrusive memories, for example, by competition for limited cognitive resources. By deriving a time-inhomogeneous version of the stochastic model (see electronic supplementary material), different combinations of intervention components can be investigated. Delivering a single dose of task in the first time period, allows us to evaluate the long-term probability of memories successfully being rendered non-intrusive or returning to an intrusive memory state (figure 3*a*). Increasing task strengths decrease the probability of intrusive memories remaining unaltered.

Delivering a single dose of task in the first time period has greater marginal gains in reducing the probability of intrusive memories reduction than no task interventions (figure 3*b*). However, over time, these differences reduce, and altering the task or task parameters might be necessary to prevent the intrusive memory reoccurring. Combining multiple tasks (say, two types of behavioural tasks) that act synergistically (additively or multiplicatively) can have greater effect at further reducing the probability of intrusive memories reoccurring. Delivering multiple doses of task(s) in the first time period is expected to achieve greater reductions in patterns of intrusive memories occurring than single tasks (figure 3*c*).

**Figure 5.**
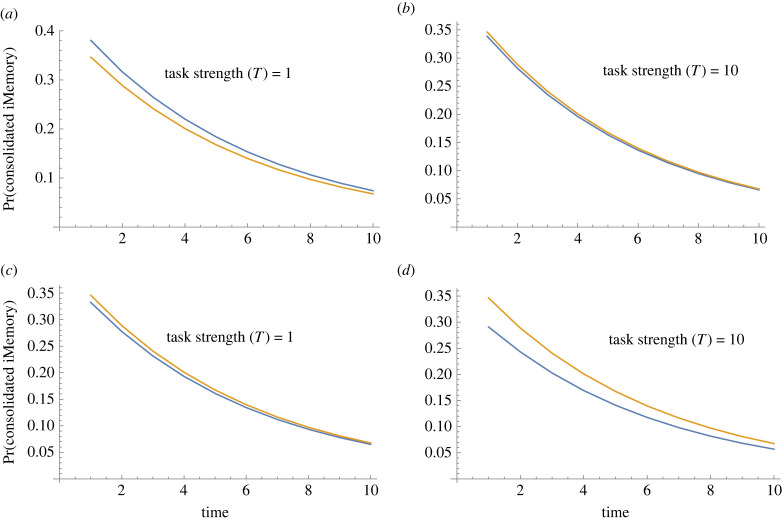
Simulation outcomes of trauma memory model for the effects of reminder cue on the probability of intrusive memories (iM) reconsolidating. (*a,b*) Outcome on the probability that iMs reconsolidate vary with multiple independent reminder cues and task strengths. (*a*) When the task strength is low (*T* = 1), multiple reminder cues increase likelihood of intrusive memories reconsolidating (task blue line; no-task orange line). (*b*) When task strength is high (*T* = 10), multiple reminder cues interact to affect the efficacy of the task (no difference between task/no task outcomes). Multiple reminders weaken task interventions in prevent iMs reconsolidating. (*c,d*) By contrast, under conditionally dependent reminder cues (where the strength of cue weakens compared with the magnitude of the previous cue), then there is no interaction between task and reminder cue. Reminder cue together with the intervention task can reduce the probability of intrusive memories reconsolidating (task blue line; no-task orange line).

Multiple independent memory reminder cue events (*p*_3_*^n^*; where *n* is the number of reminder cue events) interact with task strength to affect the probability of intrusive memories. Delivering multiple reminder cues under different task intervention can affect intrusive memory reoccurrence (figure 3*a,b*). Critically, under weak task interventions, multiple reminder cues can increase the likelihood of intrusive memories reoccurring (figure 5*a,b*) and thus worsen symptoms. Coupled with high probability of intrusive memories reconsolidating into their original form (*p*_5_), multiple reminder cues acting independently reduce the probability of reactivation (*p*_3_) and increase intrusive memory reoccurrence. By contrast, with conditionally dependent reminder events (whereby the strength of subsequent reminder cues weakens compared with the strength of the previous reminder cue), then there is no interaction between task intervention and reminder cue; task interventions act to reduce the probability of intrusive memories reoccurring (figure 5*c,d*) and may improve symptoms.

**Table 1.** Explanation of key terms and essential mathematical notation.

	definition	notes
key terms		
**iM**	intrusive memory	a recurrent memory that flashes back (involuntarily) into the mind's eye (mental imagery), e.g. vivid visual scene from a traumatic event
		unwanted intrusive memory recall (rather than deliberately recalled form of the memory) is central to clinical posttraumatic stress
**niM**	non-intrusive form of memory	a memory of the same event that does not come to mind involuntarily, (but could be deliberately recalled)
consolidation	processes by which memories form	after experiencing an event, there is a period of time while the memory is malleable before being stored (or not) in longer term memory
reactivation	processes by which memories are recalled and made malleable	while it is malleable, the reactivated memory can be updated—weakened or strengthened (or unchanged)^a^
reconsolidation	process whereby reactivation of a previously consolidated memory renders it malleable; restabilization is then required for the memory to persist^a^	reconsolidation offers a mechanism through which memory can be modified (strengthened or weakened); it provides a framework to generate hypotheses about memory updating
reminder cue	intervention component *to* reactivate memories	for reconsolidation to occur, a memory must be reactivated via a retrieval cue^a^
task	intervention *on* reactivated memories to make them non-intrusive	these tasks can be pharmacological, physical or behavioural
visuospatial task	interventions that interfere with holding a visual mental image in mind^a^	playing Tetris® is one example
essential mathematical notation		
**P**	transition matrix	an array used to define the Markov chain that includes both the reminder cue and the task intervention
**Q**	transition matrix	an array used to define the Markov chain without the reminder cue and the task intervention
*p_i_*	transition probabilities	probabilities describing memories changing from one state to another
*T*	task strength	in the expression *p*5 = 1/(1 + *T*) (equation (2.2)), *T* describes the magnitude of the task affecting the transition from reactivated memory to consolidated iM (figure 1)
*α*	reactivation cue strength	in the expression, *p*_3_ = 1/(1 + exp(−*α*)) (equation (2.3)), *α* describes the magnitude of the reactivation cue affecting the transition from consolidated iM state to reactivated memory sate
*π*	state vector	a vector used to describe the distribution of memories in different states
*n*	time steps	use to iterate the Markov chain and determine steady states
*λ*	eigenvalues	scalar values derived from the transition matrix; uses to define ‘mixing times’ (see electronic supplementary material equations A16–A18)
*τ*	time to convergence	measure of time before memories consolidate into the non-intrusive state

^a^Definitions are taken from [28].

## 4. Discussion

Here, we have introduced a quantitative framework for understanding the modification of intrusive memories after traumatic events, and how targeting them in an intervention may help make them become less intrusive. We show how coupling reminder cues and task strengths can both influence the likelihood of reducing the reoccurrence of intrusive memories, as can their combination. We show, empirically, how the model framework can be used to evaluate the success of interventions and the key model sensitivities that allow intrusive memories to persist. Critical to this, the model framework developed here provides a predictive approach to understanding components of treatment interventions (e.g. task doses, reminder cue frequency) which have important clinical implications.

### 4.1. Memory reactivation strength and frequency matters

Maintenance of different memories is affected by reactivation cues (e.g. [7]). For example, single presentations of a conditioned stimulus can induce reconsolidation and influence memory persistence. However, multiple cues can disrupt memories and lead to loss of acquired conditioned responses [7]. Here, in this study, we have shown that the number of repeated memory reminder cues affects memory persistence, and multiple independent reactivation cues can render iMs *more* intrusive (for example multiple reminder cues can weaken task interventions, figure 3). By contrast, *weakening* multiple reactivation cues can reduce the probability that reactivated, labile iMs reconsolidate.

Further to understanding memory reactivation and memory lability is how the strength of the cues can weaken or strengthen a memory. For instance, moderate levels of memory (re)activation are argued to be sufficient to lead to forgetting a memory [33]. Under a no-think/think paradigm, a non-monotonic relationship exists between memory activation and the consequential strength of the memory [33]: weak activation has a limited effect on weakening a memory; moderate activation has optimal effect of memory weakening; while strong activation can strengthen the memory. Moreover, *incomplete* reminder cues which lead to prediction errors (differences between prior learned experience and a contemporary reality) allow memories to be destabilized, become labile and modified [34]. Here, we find that *weakening* sequential reminder cues can reduce intrusive memories: further investigating how pre-existing expectations, the type of the reminder cue and intrusive memory reactivation lead to new learning, memory encoding and reinforcement necessitates future study. Overall, and perhaps counterintuitively clinically, weaker reminder cues are predicted to be more effective than stronger cues.

A corollary of all this is that intrusive memories operate within networks of brain architecture—changes in the amygdala, hippocampus and pre-frontal cortex occur following traumatic events [35]. Using real-time neural measures allows loops and networks across brain activity to be visualized [36]. So, if the strength or number of reactivation cues lead to nonlinear patterns in the changes to the reconsolidation of an intrusive memory and/or its reduction in intrusiveness by reconsolidation of a neutral memory, then further study, extending the Markov chain framework we develop here, is clearly warranted. Network-level effects of competition between iMs and niMs, the disruption of intrusive memory reconsolidation across an emotional-memory network and how information on consolidation/reconsolidation flows through these sorts of networks are all amenable questions within the stochastic modelling framework developed here.

Our framework suggests that briefer memory reactivation cue durations without multiple repetitions would be preferable for treatment success in reducing the number of intrusive memories. This is of key clinical interest as current evidence-based psychological treatments [37] involve deliberately recalling the trauma memory often in a prolonged (and repeated) way, which, while a form of treatment in itself, can be aversive and lead to patient dropout [38,39]. Shortening the duration of the memory reactivation cue may not only help make treatment more effective but also more tolerable for patients and could increase successful completion rates in therapy. The quality of memory reminder cues to achieve memory reactivation and adaptive memory updating requires calibration and may draw on insights from non-trauma memory [40].

Furthermore, our framework suggests increasing the strength of the intervention task procedure is associated with poorer outcomes (see figure 2). That is, increasing the strength of the task (here, the visuospatial task Tetris® gameplay) reduces the chance of intrusive memories reconsolidating into a non-intrusive memory state and can lead to trauma memories continuing to be intrusive. Many of those delivering clinical treatments and/or support after trauma might assume that conducting a longer and more intense treatment procedure(s) (here modelled as task strength) would be better than shorter ones. Our results suggest the reverse: decreasing the strength task procedure is associated with more beneficial outcomes in reducing the number of intrusive memories. Overall, this opens the intriguing possibility of optimizing mental health treatments via research-driven insights from a mechanistically driven, quantitative framework, rather than relying solely on practice-driven conventions that continue to dominate mental health research. To eliminate the recurrence of intrusive memories it may be optimal to use briefer and more focused procedures targeting one intrusive memory at a time, rather than long and intense sessions reliving a whole trauma episode.

### 4.2. Task interventions can influence suites of memory states

Following memory reactivation, both pharmacological and non-pharmacological interventions can interfere with memories. Studies have shown how different interventions influence memory states [7]. Here we show that interventions, tacitly through non-pharmacological approaches [28,41], can determine times memories are in an intrusive state and as task strength increases, the time before memories enter a neutral state. For many people, intrusive memories following trauma might weaken over time without intervention [42–44]. However, for some they do not, so these sorts of interventions can be highly beneficial. Laboratory and clinical studies have shown that treatment interventions with a cognitive task can reduce the propensity of the intrusive memories to (re)consolidate following a memory reminder cue soon after a trauma [28–31,45]. Furthermore, there is emerging evidence for the success of these interventions when delivered at later time intervals since the trauma occurred [32,46,47]. Here, we have shown that delivering multiple doses or different (task) interventions is likely to achieve greater marginal gains in reducing the probability of intrusive memories reconsolidating than simply increasing the strength of a single-task intervention.

### 4.3. Bounds on outcomes

General and empirically derived predictions from the stochastic framework highlight that there are bounds on memory reconsolidation outcomes following reactivation. Different combinations of task intervention strength and reactivation cue strength can lead to the same outcome in minimizing the time before memories consolidate into a non-intrusive state. However, bounds exist on the time taken for memories to enter this state and these are critically dependent on the length of the reconsolidation window. Understanding the critical time constraints on optimizing outcomes may require incorporating the details of neural circuitry dynamics (to understanding how neurons inhibit and excite to influence the length of the reconsolidation window) together with the time required to interrupt intrusive emotional memories with competing tasks.

Furthermore, empirical validation of this stochastic framework against experimental or clinical data will require the formulation of appropriate likelihood frameworks [48,49]. As shown here, cross-sectional designed experiments can allow canonical transition probabilities to be determined. With longitudinal data and appropriate consideration of the time series correlative structures, statistically validating non-homogeneous transition matrices will be feasible—this will allow evaluation the ongoing temporal success of reactivation probabilities, the lability of the memories, task interventions and the consolidation into a non-intrusive state.

### 4.4. Framework for testing cognitive models of traumatic memory

Here, we have introduced a framework that is distinct in that it provides a conceptual way to model the processes of so-called memory consolidation and reconsolidation. It is amenable to direct parametrization from experiments and has the value to be used as a part of clinical tools for the assessment and evaluation of interventions aimed at reducing the persistence of intrusive memories after traumatic events.

A unique advantage of our quantitative framework is that it links cognitive perspectives of trauma to the processes of memory reconsolidation. While cognitive conceptual models for the implications of trauma are well developed [20–23], they lack the mechanistic detail we develop here. These cognitive conceptual models are underpinned by the memory of the trauma being characterized by the frequency of involuntary intrusive memories. Early social-cognitive models such as Horowitz's formulation of a stress-response syndrome [20] focus on the interplay between completion tendency (integrating trauma information on acceptable cognitive world model) and psychological defences to keep the trauma information in an unconscious state; it is then this oscillation between integrating trauma and psychological defences that lead to flashbacks and intrusions. Critically, Horowitz's formulation emphasizes the dynamic nature of trauma memory consolidation. Through our framework, this cognitive model is directly amenable to testing through an understanding of the probability by which memories consolidate—here we have assumed that trauma always leads to intrusive memory consolidate (*p*_1_ → 1.0). However, that need not be the case and building more dynamic, information-processing structures into the consolidation of trauma memory will allow different cognitive models of traumatic memory to be validated.

Under alternative conceptual frameworks, versions of the so-called dual representation theory [24,50] posit that intrusive memories occur as an imbalance between the strengthening of emotion-laden sensory-bound representations and weakening of contextual representations in which the traumatic event occurred. Either strengthening of self-to-object (egocentric) and/or weakening of object-to-object (allocentric) memory processing can lead to the development of more intrusive memories [25,51,52]. The framework we develop here investigates the memory reconsolidation processes associated with changes in allocentric memory effects. Straightforward extensions of the mathematical framework, coupling different stochastic Markov chains, developed in this study, could allow versions of the dual representation theory to be parametrized. These coupled Markov chains could then allow predictions of both egocentric and allocentric memory processing of traumatic events to be compared and contrasted. Together with the mathematical approaches developed in our work here and elsewhere [26,27], this may allow a way to combine mechanistic neural detail and cognitive process for a greater understanding of mental health disorders.

## 5. Conclusion

Here, we have shown that both the number of reactivation cues and the strength of the intervention tasks influence the outcome of intrusive memories persisting, and that their combination can also be important.

The same-strength (independent), multiple reactivation cues can lead to trauma memories being more intrusive (i.e. worsening any possible treatment outcomes). Tapering the strength of reactivation cues, by making them weaker, can reduce the probability of memories being intrusive. Cues could be weakened for example by making them briefer or decreasing the cue strength in some way such as making the memory less vivid, intense and/or emotional.

Increasing the strength of the intervention task reduces the chance of intrusive memories reconsolidating into a non-intrusive memory state and can lead to trauma memories continuing to be intrusive. Furthermore, multiples tasks that are designed to act synergistically can reduce the probability of intrusive memories reoccurring. Examples of synergistic tasks in the intervention paradigm discussed here include a digital visuospatial computer game but could also be a physical visuospatial intervention such as clay modelling, or possibly a synergistic task in another modality such as targeted neurostimulation.

Combinations of both treatment tasks and reactivation cues affect the reoccurrence of intrusive memories: weak tasks together with multiple (same-strength) reminder cues increase the occurrence of intrusive memories. However, if the strength of the reminder cues taper and weaken, task interventions act to decrease the reoccurrence of intrusive memories.

Together with themes presented elsewhere [26,27,53], the stochastic modelling approach developed here provides a hierarchical, mechacognitive framework in which it is now feasible to embed neural mechanisms and cognitive processes. The fact that the stochastic framework opens up a set of new ideas, predictions and outcomes, and provides a unique way in which to explore memory updating (e.g. via consolidation and reconsolidation) is compelling for further developments [54]. Predictions (albeit counterintuitive clinically) that less reactivation strength for the memory reminder cue and weaker task strengths favour a reduction in intrusive memories is intriguing.

The impact of traumatic events on memory is to create a (limited) number of *different* intrusive memories, so-called hotspots [55,54]. These forms of memory are amenable to similar intervention after different types of traumatic situations (road traffic accidents; traumatic childbirths; work-related trauma of intensive care unit staff etc.). This underscores the continued need for empirical support for mechanistically driven, quantitative frameworks to extend our understanding of the temporal dynamic processes of treatments to reduce the persistence of intrusive memories after trauma.

## Data Availability

All analyses were completed in Mathematica and the scripts are available from the Open Science Framework: https://osf.io/v4ynf/. The data are provided in the electronic supplementary material [56].

## References

[1] Kilpatrick DG, Resnick HS, Milanak ME, Miller MW, Keyes KM, Friedman MJ. 2013 National estimates of exposure to traumatic events and PTSD prevalence using DSM-IV and DSM-5 criteria. J. Trauma. Stress **26**, 537-547. (10.1002/jts.21848) 24151000 PMC4096796

[2] American Psychiatric Association. 2013 Diagnostic and statistical manual of mental disorders, 5th edn. Arlington, VA: American Psychiatric Association.

[3] McGaugh JL. 2013 Marking lasting memories: remembering the significant. Proc. Natl Acad. Sci. USA **110**, 10 402-10 407. (10.1073/pnas.1301209110) PMC3690616 23754441

[4] Tulving E. 1972 Episodic and semantic memory. In Organization of memory (eds E Tulving, W Donaldson), pp. 381-403. New York, NY: Academic Press.

[5] Bisson JI et al. 2019 The international society for traumatic stress studies new guidelines for the prevention and treatment of posttraumatic stress disorder: methodology and development process. J. Trauma. Stress **32**, 475-483. (10.1002/jts.22421) 31283056

[6] Gräff J et al. 2014 Epigenetic priming of memory updating during reconsolidation to attenuate remote fear memories. Cell **156**, 261-276. (10.1016/j.cell.2013.12.020) 24439381 PMC3986862

[7] Merlo E, Milton AL, Goozée ZY, Theobald DE, Everitt BJ. 2014 Reconsolidation and extinction are dissociable and mutually exclusive processes: behavioral and molecular evidence. J. Neurosci. **34**, 2422-2431. (10.1523/JNEUROSCI.4001-13.2014) 24523532 PMC3921417

[8] Monfils MH, Holmes EA. 2018 Memory boundaries: opening a window inspired by reconsolidation to treat anxiety, trauma-related and addiction disorders. Lancet Psychiat. **5**, 1032-1042. (10.1016/S2215-0366(18)30270-0) 30385214

[9] McGaugh JL. 2000 Memory—a century of consolidation. Science **287**, 248-251. (10.1126/science.287.5451.248) 10634773

[10] Moscovitch M, Nadel L. 1998 Consolidation and the hippocampal complex revisited: in defense of the multiple-trace model. Curr. Opin. Neurobiol. **8**, 297-300. (10.1016/S0959-4388(98)80155-4) 9635217

[11] Alberini CM, LeDoux JE. 2013 Memory reconsolidation. Curr. Biol. **23**, R746-R750. (10.1016/j.cub.2013.06.046) 24028957

[12] Sara SJ. 2000 Retrieval and reconsolidation: toward a neurobiology of remembering. Learn. Mem. **7**, 73-84. (10.1101/lm.7.2.73) 10753974

[13] Nader K, Schafe GE, Le Doux JE. 2000 Fear memories require protein synthesis in the amygdala for reconsolidation after retrieval. Nature **406**, 722-726. (10.1038/35021052) 10963596

[14] Forcato C, Burgos VL, Argibay PF, Molina VA, Pedreira ME, Maldonado H. 2007 Reconsolidation of declarative memory in humans. Learn. Mem. **14**, 295-303. (10.1101/lm.486107) 17522018 PMC2216535

[15] Monfils MH, Cowansage KK, Klann E, LeDoux JE. 2009 Extinction-reconsolidation boundaries: key to persistent attenuation of fear memories. Science **324**, 951-955. (10.1126/science.1167975) 19342552 PMC3625935

[16] Elsey JW, van Ast VA, Kindt M. 2018 Human memory reconsolidation: a guiding framework and critical review of the evidence. Psychol. Bull. **144**, 797. (10.1037/bul0000152) 29792441

[17] Beckers T, Kindt M. 2017 Memory reconsolidation interference as an emerging treatment for emotional disorders: strengths, limitations, challenges, and opportunities. Annu. Rev. Clin. Psychol. **13**, 99-121. (10.1146/annurev-clinpsy-032816-045209) 28375725 PMC5424072

[18] Lee JL. 2009 Reconsolidation: maintaining memory relevance. Trends. Neurosci. **32**, 413-420. (10.1016/j.tins.2009.05.002) 19640595 PMC3650827

[19] Astill Wright L, Horstmann L, Holmes EA, Bisson JI. 2021 Consolidation/reconsolidation therapies for the prevention and treatment of PTSD and re-experiencing: a systematic review and meta-analysis. Transl. Psychiat. **11**, 1-14. (10.1038/s41398-021-01570-w) PMC8417130 34480016

[20] Horowitz MJ. 1976 Stress response syndromes. New York, NY: Jason Aronson.

[21] Brewin CR, Dalgleish T, Joseph S. 1996 A dual representation theory of posttraumatic stress disorder. Psychol. Rev. **103**, 670-686. (10.1037/0033-295X.103.4.670) 8888651

[22] Ehlers A, Clark DM. 2000 A cognitive model of posttraumatic stress disorder. Behav. Res. Ther. **38**, 319-345. (10.1016/S0005-7967(99)00123-0) 10761279

[23] Brewin CR, Holmes EA. 2003 Psychological theories of posttraumatic stress disorder. Clin. Psychol. Rev. **23**, 339-376. (10.1016/S0272-7358(03)00033-3) 12729677

[24] Brewin CR, Gregory JD, Lipton M, Burgess N. 2010 Intrusive images in psychological disorders: characteristics, neural mechanisms, and treatment implications. Psychol. Rev. **117**, 210-232. (10.1037/a0018113) 20063969 PMC2834572

[25] Bisby JA, Burgess N, Brewin CR. 2020 Reduced memory coherence for negative events and its relationship to posttraumatic stress disorder. Curr. Dir. Psychol. Sci. **29**, 267-272. (10.1177/0963721420917691) 33214741 PMC7643751

[26] Bonsall MB, Geddes JR, Goodwin GM, Holmes EA. 2015 Bipolar disorder dynamics: affective instabilities, relaxation oscillations and noise. J. R. Soc. Interface **12**, 20150670. (10.1098/rsif.2015.0670) 26577592 PMC4685840

[27] Holmes EA et al. 2018 The Lancet psychiatry commission on psychological treatments research in tomorrow's science. Lancet Psychiat. **5**, 237-286. (10.1016/S2215-0366(17)30513-8) 29482764

[28] James EL, Bonsall MB, Hoppitt L, Tunbridge EM, Geddes JR, Milton AL, Holmes EA. 2015 Computer game play reduces intrusive memories of experimental trauma via reconsolidation update mechanisms. Psychol. Sci. **26**, 1201-2015. (10.1177/0956797615583071) 26133572 PMC4526368

[29] Singh L, Espinosa L, Ji JL, Moulds ML, Holmes EA. 2020 Developing thinking around mental health science: the example of intrusive, emotional mental imagery after psychological trauma. Cogn. Neuropsychiatry **25**, 348-363. (10.1080/13546805.2020.1804845) 32847486

[30] Iyadurai L, Blackwell SE, Meiser-Stedman R, Watson PC, Bonsall MB, Geddes JR, Nobre AC, Holmes EA. 2018 Preventing intrusive memories after trauma via a brief intervention involving Tetris computer game play in the emergency department: a proof-of-concept randomized controlled trial. Mol. Psychiat. **23**, 674-682. (10.1038/mp.2017.23) PMC5822451 28348380

[31] Kanstrup M, Singh L, Göransson KE, Widoff J, Taylor RS, Gamble B, Iyadurai L, Moulds ML, Holmes EA. 2021 Reducing intrusive memories after trauma via a brief cognitive task intervention in the hospital emergency department: an exploratory pilot randomised controlled trial. Transl. Psychiatry **11**, 30. (10.1038/s41398-020-01124-6) 33431807 PMC7798383

[32] Ramineni V, Millroth P, Iyadurai L, Jaki T, Kingslake J, Highfield J, Summers C, Bonsall MB, Holmes EA. 2023 Treating intrusive memories after trauma in healthcare workers: a Bayesian adaptive randomized trial developing an imagery-competing task intervention. Mol. Psychiatry **26**, 1-10. (10.1038/s41380-023-02062-7) PMC10131522 37100869

[33] Detre GJ, Natarajan A, Gershman SJ, Norman KA. 2013 Moderate levels of activation lead to forgetting in the think/no-think paradigm. Neuropsychologia **51**, 23 711-22 388. (10.1016/j.neuropsychologia.2013.02.017) PMC3702674 23499722

[34] Sinclair A, Barense MD. 2019 Prediction error and memory reactivation: how incomplete reminders drive reconsolidation. Trends Neurosci. **42**, 727-739. (10.1016/j.tins.2019.08.007) 31506189

[35] Brenmer JD. 2006 The relationship between cognitive and brain changes in posttraumatic stress disorder. Ann. NY Acad. Sci. **1071**, 80-86. (10.1196/annals.1364.008) 16891564 PMC3233753

[36] deBettencourt MT, Turk-Browne NB, Norman KA. 2019 Neurofeedback helps to reveal a relationship between context reinstatement and memory retrieval. Neuroimage **200**, 292-301. (10.1016/j.neuroimage.2019.06.001) 31201985 PMC7034791

[37] Lewis C, Roberts NP, Andrew M, Starling E, Bisson J. 2020 Psychological therapies for post-traumatic stress disorder in adults: systematic review and meta-analysis. Eur. J. Psychotraumatol. **11**, 1729633. (10.1080/20008198.2020.1729633) 32284821 PMC7144187

[38] Lewis C, Roberts NP, Gibson S, Bisson JI. 2020 Dropout from psychological therapies for post-traumatic stress disorder (PTSD) in adults: systematic review and meta-analysis. Eur. J. Psychotraumatol. **11**, 1709709. (10.1080/20008198.2019.1709709) 32284816 PMC7144189

[39] Foa EB et al. 2018 STRONG STAR Consortium. Effect of prolonged exposure therapy delivered over 2 weeks vs 8 weeks vs present-centered therapy on PTSD symptom severity in military personnel: a randomized clinical trial. JAMA **319**, 354-364. (10.1001/jama.2017.21242) 29362795 PMC5833566

[40] St. Jacques PL, Olm C, Schacter DL. 2013 Neural mechanisms of reactivation-induced updating that enhance and distort memory. Proc. Natl Acad. Sci. USA **110**, 19 671-19 678. (10.1073/pnas.1319630110) PMC3856820 24191059

[41] Kredlow MA, Otto MW. 2015 Interference with the reconsolidation of trauma-related memories in adults. Depress. Anxiety **32**, 32-37. (10.1002/da.22343) 25585535

[42] O'Donnell ML, Elliott P, Lau W, Creamer M. 2007 PTSD symptom trajectories: from early to chronic response. Behav. Res. Ther. **45**, 601-606. (10.1016/j.brat.2006.03.015) 16712783

[43] Galatzer-Levy IR, Karstoft KI, Statnikov A, Shalev AY. 2014 Quantitative forecasting of PTSD from early trauma responses: a machine learning application. J. Psychiatr. Res. **59**, 68-76. (10.1016/j.jpsychires.2014.08.017) 25260752 PMC4252741

[44] Galatzer-Levy IR, Huang SH, Bonanno GA. 2018 Trajectories of resilience and dysfunction following potential trauma: a review and statistical evaluation. Clin. Psychol. Rev. **63**, 41-55. (10.1016/j.cpr.2018.05.008) 29902711

[45] Horsch A, Yvan V, Favrod C, Morisod Harari M, Blackwell SE, Watson P, Iyadurai L, Bonsall MB, Holmes EA. 2017 Reducing intrusive traumatic memories after emergency caesarean section: a proof-of-principle randomized controlled study. Behav. Res. Therapy **94**, 36-47. (10.1016/j.brat.2017.03.018) PMC5466064 28453969

[46] Kessler H, Holmes EA, Blackwell SE, Schmidt AC, Schweer JM, Bücker A, Herpertz S, Axmacher N, Kehyayan A. 2018 Reducing intrusive memories of trauma using a visuospatial interference intervention with inpatients with posttraumatic stress disorder (PTSD). J. Consult. Clin. Psychol. **86**, 1076-1090. (10.1037/ccp0000340) 30507232

[47] Deforges C, Fort D, Stuijfzand S, Holmes EA, Horsch A. 2022 Reducing childbirth-related intrusive memories and PTSD symptoms via a single-session behavioural intervention including a visuospatial task: a proof-of-principle study. J. Affect. Disord. **303**, 64-73. (10.1016/j.jad.2022.01.108) 35108604

[48] Edwards AWF. 1990 Likelihood. Baltimore, MD: John Hopkins University Press.

[49] Morgan BJT. 2008 Applied stochastic modelling, 2nd edn. London, UK: Chapman & Hall.

[50] Brewin CR. 2014 Episodic memory, perceptual memory, and their interaction: foundations for a theory of posttraumatic stress disorder. Psychol. Bull. **140**, 69-97. (10.1037/a0033722) 23914721

[51] Bisby JA, King JA, Brewin CR, Burgess N, Curran HV. 2010 Acute effects of alcohol on intrusive memory development and viewpoint dependence in spatial memory support a dual representation model. Biol. Psychiatry **68**, 280-286. (10.1016/j.biopsych.2010.01.010) 20202625

[52] Sierk A, Manthey A, King J, Brewin CR, Bisby JA, Walter H, Burgess N, Daniels JK. 2019 Allocentric spatial memory performance predicts intrusive memory severity in posttraumatic stress disorder. Neurobiol. Learn. Mem. **166**, 107093. (10.1016/j.nlm.2019.107093) 31536787

[53] Holmes EA, Espinosa L, Visser RM, Bonsall MB, Singh L. 2020 Psychological interventions as they relate to intrusive thinking: the example of intrusive, emotional mental imagery. In Intrusive thinking: from molecules to free will (eds PW Kalivas, MP Paulus), pp. 287-313. Cambridge, MA: MIT Press.

[54] Singh L, Garate B, Hoppe JM, Holmes EA. 2022 Qualitative analysis of hotspots and intrusive memories after viewing an aversive film highlights their sensory and spatial features. Sci. Rep. **12**, 7049. (10.1038/s41598-022-10579-0) 35487945 PMC9052176

[55] Hoppe JM, Walldén YS, Kanstrup M, Singh L, Agren T, Holmes EA, Moulds ML. 2022 Hotspots in the immediate aftermath of trauma – mental imagery of worst moments highlighting time, space and motion. Conscious Cogn. **99**, 103286. (10.1016/j.concog.2022.103286) 35220032

[56] Bonsall MB, Holmes EA. 2023 Temporal dynamics of trauma memory persistence. Figshare. (10.6084/m9.figshare.c.6662671)

